# LC-HRMS/MS-Based Metabolomics Approaches Applied to the Detection of Antifungal Compounds and a Metabolic Dynamic Assessment of Orchidaceae

**DOI:** 10.3390/molecules27227937

**Published:** 2022-11-16

**Authors:** Gesiane S. Lima, Nerilson M. Lima, Jussara V. Roque, Deborah V. A. de Aguiar, João V. A. Oliveira, Gabriel F. dos Santos, Andrea R. Chaves, Boniek G. Vaz

**Affiliations:** Institute of Chemistry, Federal University of Goias, Goiania 74690-900, GO, Brazil

**Keywords:** liquid chromatography–mass spectrometry, untargeted metabolomics, metabolic dynamic, antifungal compounds

## Abstract

The liquid chromatography–mass spectrometry (LC-MS)-based metabolomics approach is a powerful technology for discovering novel biologically active molecules. In this study, we investigated the metabolic profiling of Orchidaceae species using LC-HRMS/MS data combined with chemometric methods and dereplication tools to discover antifungal compounds. We analyze twenty ethanolic plant extracts from *Vanda* and *Cattleya* (Orchidaceae) genera. Molecular networking and chemometric methods were used to discriminate ions that differentiate healthy and fungal-infected plant samples. Fifty-three metabolites were rapidly annotated through spectral library matching and in silico fragmentation tools. The metabolomic profiling showed a large production of polyphenols, including flavonoids, phenolic acids, chromones, stilbenoids, and tannins, which varied in relative abundance across species. Considering the presence and abundance of metabolites in both groups of samples, we can infer that these constituents are associated with biochemical responses to microbial attacks. In addition, we evaluated the metabolic dynamic through the synthesis of stilbenoids in fungal-infected plants. The tricin derivative flavonoid- and the loliolide terpenoidfound only in healthy plant samples, are promising antifungal metabolites. LC-HRMS/MS, combined with state-of-the-art tools, proved to be a rapid and reliable technique for fingerprinting medicinal plants and discovering new hits and leads.

## 1. Introduction

Orchidaceae is one of the largest and most diverse families in the plant kingdom, with more than 28,000 species and 763 genera [1,2]. Orchidaceae species, popularly known as orchids, are sold commercially for presenting beautiful and diverse flowers and the economically important spice known as vanilla [3]. Many species have been studied for their chemical composition and pharmacological activities, including anticancer, anti-inflammatory, antioxidant, neuroprotective, antivirus, and antimicrobial [3]. Several orchid species are known to produce stilbenoids, phytoalexins responsible for protection against predation and antimicrobial activities [4,5,6]. Orchinol and hircinol are two stilbenoid compounds isolated from the *Orchis* and *Loroglossum* genera, respectively, which were reported with antifungal activity and play a role in the defense of orchid tubes [7].

Considering the antifungal activity of the secondary metabolites present in orchids and the low rate of new drug discovery, there is an urgent need to develop fast and efficient methodologies for screening new antimicrobial agents. Since the classic process of screening bioactive natural products is a time-consuming and labor-intensive step, analytical techniques applied to separating and characterizing phytocompounds stand out for their high ability to dereplicate complex samples and detect constituents even at low concentrations that can be useful in planning new drugs [8,9].

Mass spectrometry (MS)-based analytical platforms are established as the technique of choice in metabolomic investigations due to their high selectivity, sensitivity, speed, and versatility in detecting a wide range of analytes with different physicochemical properties. In addition, the development of increasingly sensitive instruments allows the detection of trace-level molecules and provides analysis with sub-ppm mass accuracy that allows describing the molecular formula with high precision based on the mass defect [10,11]. In this way, the high resolving power offered by this new equipment provides essential information for reliable annotation and accurate quantification of metabolites [12]. Ultrahigh-resolution mass spectrometry hyphenated to ultra-resolution chromatographic techniques represents one of the most widely employed high-throughput screening technologies in metabolomic approaches and yields greater metabolic coverage in natural product research [11,13,14,15].

Liquid chromatography coupled with mass spectrometry (LC-MS) could offer advantages in the metabolomic analysis, such as its better resolution of the complex sample allowing a good evaluation of molecular diversity content. In addition, it provides more detailed information on the metabolic composition of the samples by some chromatographic parameters such as retention time and peak shape of the analytes, allows comparison with standards improving the confidence level in structural annotations, distinguishing isobaric and isomeric metabolites that are not resolved through the fragmentation pattern and accurate molecular mass [16,17,18,19].

Numerous applications of LC-HRMS/MS, combined with state-of-the-art tools for structural annotations (such as Classical Molecular Networking [20], Dereplicator+ [21], Network Annotation Propagation [22], and Moldiscovery [23]), are reported in the literature, and many findings obtained from their applications have boosted analytical research throughout and metabolites coverage. This approach has been widely employed to annotate flavonoids and selaginellins of the roots and shoots from *Selaginella convolute* [24]; phenolic compounds of the leaves and seeds from *Erythrina velutina* [25]; several metabolites, including four phenanthrene with the antinociceptive activity of the roots from *Laelia anceps* and *Cyrtopodium macrobulbon* [26]; several flavonoids and alkaloids of the bulbs and flowers from *Fritillaria thunbergii* [27]; various compounds of the leaves, stems and roots from *Peperomia pellucida*, including compounds annotated for the first time for the genus [28]; and unique cassaine-type diterpenoids of the leaves from *Erythrophleum suaveolens* and *E. ivorense,* which were employed to distinguish morphologically-close species [29].

Thus, the present study aimed to explore the presence and abundance of secondary metabolites in healthy and fungal-infected plants from the Orchidaceae family to select potential antimicrobial candidates and a metabolic dynamic assessment. The metabolite production of ten species of Orchidaceae belonging to the genera *Vanda* and *Cattleya* was evaluated using tandem mass spectral libraries, in silico fragmentation tools, and chemometric methods from MS data obtained by Orbitrap LC-MS.

## 2. Results

In this work, we used ultrahigh-resolution mass spectrometry coupled with liquid chromatography to investigate the metabolic dynamic of healthy and fungal-infected plants of the same botanical family to identify substances with antifungal properties. Metabolome-based antifungal screening approach was evaluated in twelve species of the Orchidaceae family by evaluating the spectral similarity of samples and applying chemometric methods. Initially, the ions were discriminated using data mining and organization tools from the GNPS—Global Natural Products Social Molecular Networking (https://ccms-ucsd.github.io/GNPSDocumentation/ (accessed on 16 August 2022) platform, and the structural annotation of the metabolites was based on accurate mass (*m*/*z*), MS/MS fragmentation pattern, chromatographic retention time, and chemotaxonomy data from Orchidaceae family. We used metadata and data mining tools according to the GNPS platform documentation, and the structural information and metabolic coverage data were inspected in detail. All library hits classified as gold (thoroughly characterized as structures), silver (compound crude extract), and bronze (partial annotation) [20] were evaluated, and chemophenetics data from the Orchidaceae family confirmed the metabolic annotation.

The structural annotation of the metabolites was confirmed through the analysis of candidates and their analogs suggested by the natural products databases, reference mass spectral libraries, and spectral similarity networks resulting in level 2 identification according to the Metabolomic Standard Initiative-MSI [30].

Lyophilized extracts from two groups of samples (healthy and fungal-infected plants) of species of the genus *Vanda* (five samples) and *Cattleya* (five samples) were submitted to the evaluation of the presence of metabolites through metabolite annotations were based on searching the experimental spectra against the GNPS spectral library using the tools Classical Molecular Networking—MN [20], Dereplicator+ [21], Network Annotation Propagation—NAP [22], Moldiscovery [23], MS2LDA [31], MolNetEnhancer [32], and analysis of chromatographic data such as retention time and UV spectra unit of absorbance. The ion abundance assessment was performed using the Feature-Based Molecular Networking—FBMN [33] tool obtained from MS/MS data in positive ionization mode (ESI(+)). Annotations with high spectral similarity were prioritized, resulting in the structural annotation of 50 compounds.

### 2.1. Comprehensive Structural Annotation of Orchidaceae Species Using Molecular Networking

To obtain hits with higher structural similarity and the biosynthetic origin within molecular families from molecular networking, the threshold for the cosine score similarity was set to 0.7.

**Figure 1:**
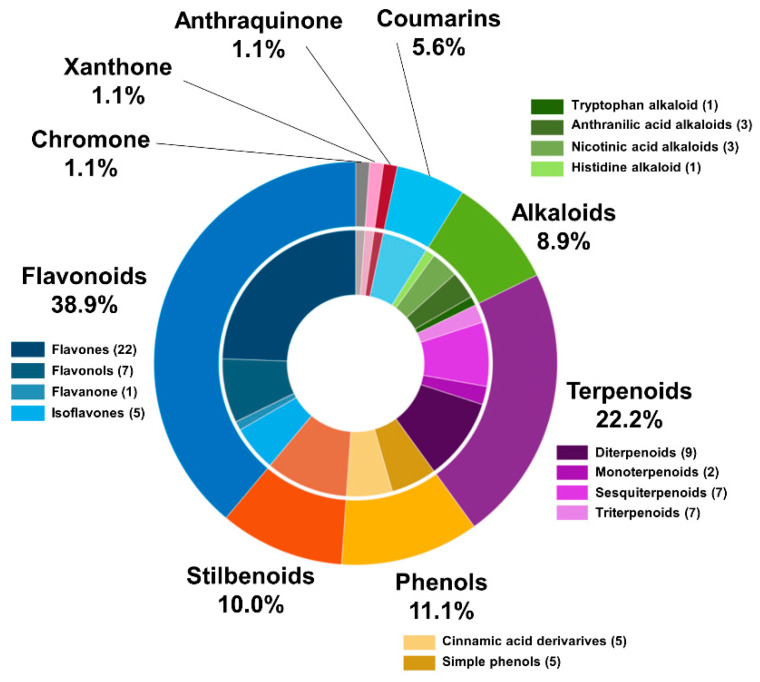
Chemical classes of the compounds annotated from *Vanda* and *Cattleya* genera obtained by LC-HRMS/MS in positive ion mode ESI.

LC-HRMS/MS-based untargeted metabolomics approaches showed a considerable variation in compounds mainly belonging to the class of secondary metabolites of the flavonoids, stilbenoids, phenolic acids, chromones, tannins, terpenoids, and steroids. Dereplication methodologies have been extensively applied in plant metabolomics and provided the structural annotation of fifty-three metabolites belonging to different classes of natural products in aglycone and heteroside form. However, most putatively annotated polyphenols were classified as heterosides, mainly glycosylated flavonoids. Among the fifty-three compounds annotated, 35 metabolites were annotated as flavonoids (22 flavones, 7 flavonols, 1 flavanone, and 5 isoflavones) and 10 stilbenoids. Regarding the phenols class, 10 compounds were annotated, being 50% cinnamic acids derivatives. Among the 20 terpenoids detected, were annotated 9 diterpenoids, 2 monoterpenoids, 7 sesquiterpenoids, and 2 triterpenoids. The library matches showed alkaloids (8) classified as tryptophan alkaloids (1), anthranilic acid alkaloids (3), nicotinic acid alkaloids (3), and histidine alkaloids (1). Other oxygenated aromatic metabolites belonging to the coumarins (5), anthraquinones (1), xanthones (1), and chromones (1) classes were found in low abundance (Figure 1). Furthermore, a wide array of primary metabolites was detected in all samples, such as carbohydrates, amino acids, and lipids.

The library matches using the classical molecular network (MN) from *Cattleya* and *Vanda* genera assessment in both physiological conditions (healthy and fungal-infected) yielded a total of 1.220 hits with 315 unique library compounds. We found 60 hits with a high confidence level, a high number of hits with gold classification, represented by spectral similarity greater than 90% (cosine score > 0.9), mz error (ppm) less than 5, and a high amount of shared peaks in the MS/MS spectrum. The analysis of the candidates suggested by the spectral library after inspection of the profile of MS/MS fragmentation patterns, evaluation of high-resolution calculation of empirical formula, and chromatographic analysis data, yielded the compounds described in Table 1. All the annotated metabolites have been previously described in the Orchidaceae family [34,35].

From the MS/MS fragmentation pattern analysis, it was observed that the *O*-glycosylated phenolic compounds were more abundant than the *C*-glycosylated compounds. *O*-glycosylated flavonoids exhibited a neutral loss of a sugar moiety, which corresponds to a loss of 162 Da for hexosides, 146 Da for deoxyhexosides and 132 Da for pentosides. The *O*-glycosylated flavonoids Rutin (C_27_H_30_O_16_), Saponarin (C_27_H_30_O_15_), Isovitexin 2″-*O*-arabinoside (C_26_H_28_O_14_), Isoschaftoside (C_26_H_28_O_14_), Tricin 5-glucoside (C_23_H_24_O_12_), Isorhamnetin 3-galactoside (C_22_H_22_O_12_), Hyperoside (C_21_H_20_O_12_), and Isovitexin (C_21_H_20_O_10_) were identified by the consecutive losses of sugar moieties, and the flavonoids aglycone Acacetin, 4′-Methoxy-6-methylflavone, and Liquiritigenin were identified by fragments originating from retro Diels–Alder reactions, and data from chromatographic analysis such as UV spectra and retention times. In addition, it was possible to perform structure-based propagation and guided detection of phenolic compounds not annotated by the spectral library. The stilbenoids Rhapontin, Erianin, 3-*O*-Methylgigantol, Gigantol, Dendrosinene B, Tristin, 3′-*O*-Methylbatatasin III, 3-[2-(3-hydroxyphenyl)ethyl]-5-methoxyphenol, and Thunalbene, metabolites found in both *Vanda* and *Cattleya* genera, were putatively annotated through accurate mass precursor and characteristic product ions such as consecutive losses of C_2_H_2_O and methoxyl groups.

**Figure 2:**
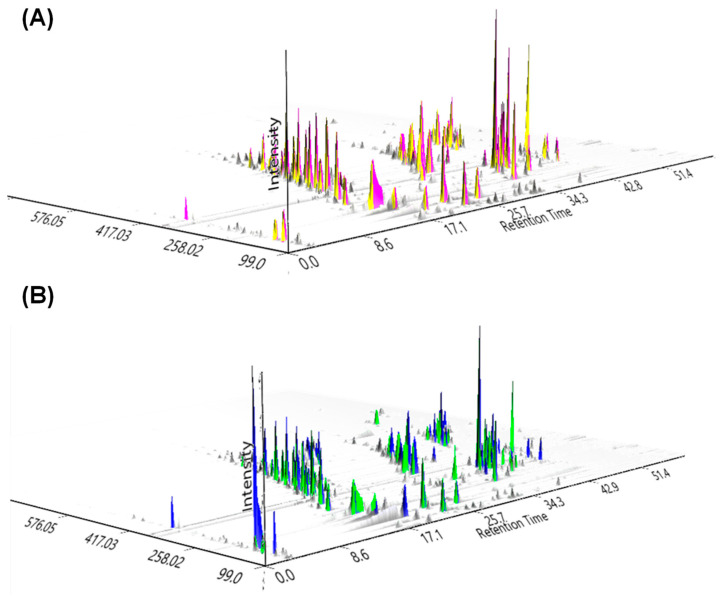
A 3D visualization of the chromatogram from (**A**) *Cattleya* fungal-infected (yellow) and healthy (pink) species; and (**B**) *Vanda* fungal-infected (green) and healthy (blue) species.

To narrow the focus to identify metabolites with antifungal potential using the strategy of investigating the metabolome of healthy plants compared with the metabolome of fungal-infected plants from the *Cattleya* and *Vanda* genera, we eliminate metabolites common to all species. The two set plants shared about 75% of their metabolome. Healthy plant species exhibited a richness of secondary metabolites (approximately 60% of hits) compared to fungal-infected plants (approximately 40% of hits), evidencing the role of these metabolites in defense against microbial attack. The attack of pathogens on plants generates a biochemical response. Therefore, the production of these chemical constituents in infected plants belonging to the same genus or species may be suppressed by environmental factors [36,37,38]. In addition, the quantitative alterations of these metabolites may also be a consequence of environmental stimuli, explaining the low abundance of some compounds in plants of the same species affected by microbiological attacks. Moreover, other factors, such as nutrient and water availability in the soil, can influence metabolic pathways and considerably affect the synthesis of secondary metabolites with antimicrobial action [39,40]. The chromatographic profile and molecular networking show subtle differences between the sample groups for both species analyzed under the same chromatographic and spectrometric conditions (Figure 2).

**Figure 3:**
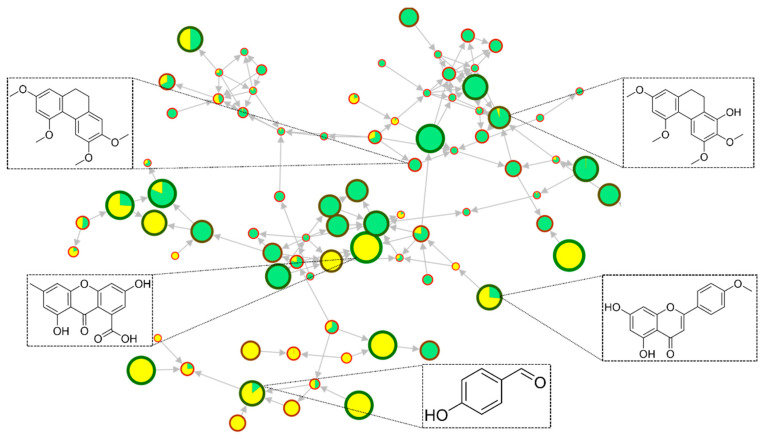
Molecular families annotated by classical molecular networking (MN) of the samples from fungal-infected plants (green) and healthy plants (yellow) and the metabolites found through GNPS library hits.

The metabolites cinnamic alcohol (phenylpropanoid), tricin 5-glucoside (flavonoid), loliolide (terpenoid), and allobetulinol (terpenoid) were detected only in samples of healthy plants. In contrast, the metabolites 3-(4-hydroxy-3-methoxyphenyl)propyl 3-(4-hydroxyphenyl)propanoate (phenol), salidroside (phenol), isovitexin (flavonoid), rutin (flavonoid), homoorientin (flavonoid), hyperoside (flavonoid), 2,3,5,7-tetramethoxy-9,10-dihydrophenanthrene (stilbenoid/phenanthrenoid), 4′-methoxy-6-methylflavone (flavonoid), isoshaftoside (flavonoid), sinapyl alcohol (phenylpropanoid), liquiritigenin (flavonoid), gigantol (stilbenoid), 3-*O*-Methylgigantol (stilbenoid), 4-methylcinnamic acid (phenylpropanoid), and isorhamnetin 3-galactoside (flavonoid) were detected only in samples of fungal-infected plants. Although the sample sets share a wide metabolic diversity, the metabolites were found at different levels of abundance since the activation of the plant defense mechanism can result in the greater activation of a biosynthesis pathway and suppress the synthesis of other metabolites. The flavonoids afrormosin, acacetin, and stilbenoids were primarily found in fungal-infected plants, while nitrogen compounds were found predominantly in samples from healthy plants. Since the infection by microorganisms induces biochemical alterations in the host organism in an attempt to defend itself from the aggressive agent [41,42,43,44], a wide diversity of metabolites was found in fungal-infected plants (Figure 3). The tricin derivative flavonoid, and the loliolide terpenoid, found only in healthy plant samples, are reported in the literature with promising antifungal activity [9,45,46].

**Figure 4:**
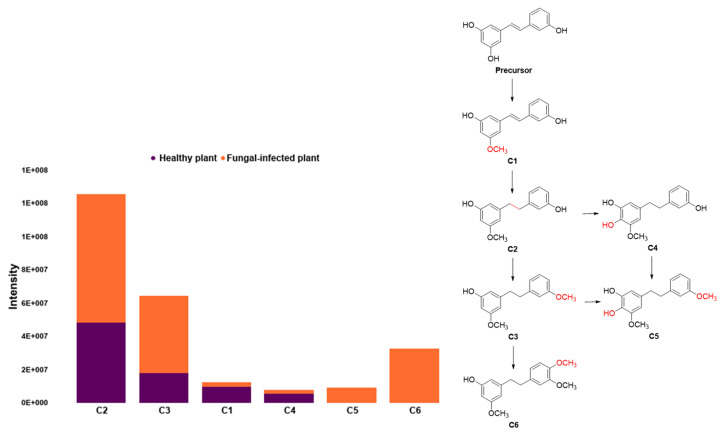
Variation of the metabolic dynamic of stilbenoids in fungal-infected plants (orange) and healthy (violet) plants.

The metabolic dynamics of Orchidaceae species were also evaluated through the synthesis of stilbenoids by fungal-infected plants and healthy plants, whose evaluation showed that the compounds, thunalbene (C1) and dendrosinene B (C4), were found in high concentration in healthy plants compared to fungal-infected plants. In contrast, the stilbenoids, batatasin III (C2) and 3′-*O*-Methylbatatasin III (C3), were found in low concentration in healthy plants compared to fungal-infected plants, while 3-Methoxy-5-[2-(3-methoxyphenyl)ethyl]-1,2-benzenediol (C5) and 3-*O*-Methylgigantol (C6) were detected only in fungal-infected plants (Figure 4). Stilbenoids are a group of plant phytoalexin polyphenols produced by plants as a defense mechanism against microbial infection [47,48].

### 2.2. Structural Annotation Strategy Using In Silico Fragmentation Tools to the Fingerprinting of Healthy and Fungal-Infected Plants from Orchidaceae

From the evaluation of the secondary metabolism of the genera *Vanda* and *Cattleya*, 145 and 202 unique metabolites were detected in samples of fungal-infected plants and healthy species from the genus *Cattleya*, respectively. For the genus *Vanda*, 146 and 166 unique metabolites were detected in fungal-infected plants and healthy species samples, respectively. The molecular diversity found in healthy plant samples evidences a greater capacity of plants to synthesize secondary metabolites with biological action. In order to obtain greater metabolic coverage, more sophisticated state-of-the-art tools were applied, such as Moldiscovery, which allows obtained structural candidates with greater accuracy and reliability. The Moldiscovery tool yielded a total of 2044 unique metabolites and 1348 unique metabolites at a cutoff score of 15 from samples of fungal-infected plants. While for samples of healthy plants, 2706 unique metabolites and 1768 unique metabolites were detected at a cutoff score of 15.

**Figure 5:**
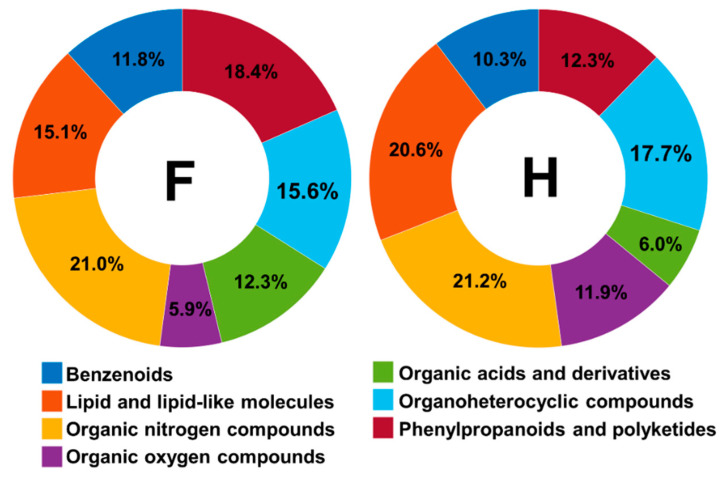
Nodes number detected through MolNetEnhancer at the “superclass” level for the samples of fungal-infected plants (F) and healthy (H) plants.

Furthermore, a greater number of unique metabolites were found in healthy plant samples (351) compared to fungal-infected plants (377) using the in silico dereplication tool termed Dereplicator+. To obtain a more comprehensive chemical overview of both sets of samples and chemical structural information, the MolNetEnhancer tool was applied to detect the chemical classes present in the samples and to assist in annotating metabolites that did not show a matching MS/MS spectrum. The analysis showed mostly a higher number of nodes for all classes at the “superclass” level for healthy plant samples, except for the classes of organic acids derivatives and phenylpropanoids and polyketides, which were more abundant in fungal-infected plants (Figure 5).

In a more comprehensive investigation at the “class” level, the coumarin derivatives were found only in healthy plant samples, while the aurone class was found only in fungal-infected plant samples. Other chemical classes were found in both sets of samples. However, they were detected in different proportions, such as stilbenes and cinnamic acid derivatives that were detected mainly in samples of fungal-infected plants, and the flavonoids, phenols, and steroids that were detected in a more significant proportion in healthy plant samples. Through molecular networking, we observe that the fungal-infected plants synthesized twice as many metabolites from the shikimate natural products pathway than healthy plants. This is because the variation of environmental conditions can silence or activate biosynthetic pathways, which impact the synthesis or reduction of the production of secondary metabolites.

**Figure 6:**
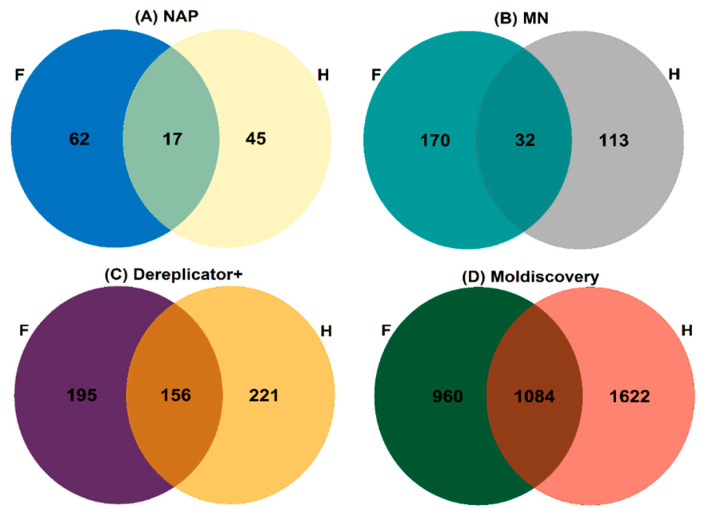
Venn diagram for the unique metabolites number and NAP-fusion annotated obtained of the workflows classical Molecular Networking (MN), Dereplicator+, Moldiscovery, and NAP from fungal-infected plants (F) and healthy (H) plants.

The propagation of structural annotations was also performed from unknown fragmentation mass spectrum analysis using the in silico tool Network Annotation Propagation (NAP), which yielded 62 metabolites putatively annotated from NAP-Fusion in silico prediction for fungal-infected plants and 45 for healthy plants samples. A Venn diagram represents the unique metabolites number and overlapped unique metabolites putatively annotated by classical Molecular Networking (MN), Dereplicator+, Moldiscovery, and NAP-fusion annotated metabolites obtained from fungal-infected plants (F) and healthy (H) plants (Figure 6).

A significant number of flavonoids and phenols were found in high abundance in healthy plant samples, which may be responsible for antimicrobial potential since they are metabolites that play a central role in plant defense against pathogen attacks [49]. The dereplication and data mining tools employed in this study provided chemical refinement of metabolomics results for exploration and guided the selection of candidates with antimicrobial potential.

### 2.3. Chemometrics Methods Applied to Orchidaceae Plants Spectral Analysis

**Figure 7:**
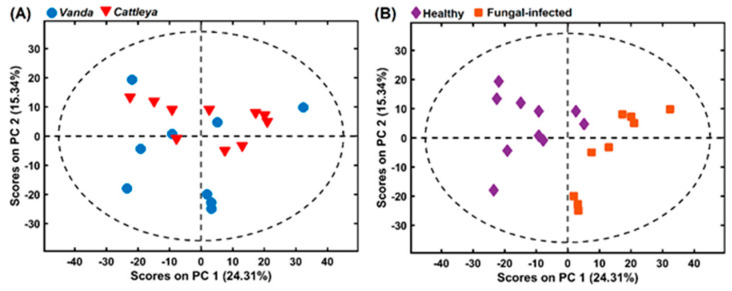
Samples visualization in 2-D space using scores from PCA with (**A**) *Vanda* and *Cattleya* and (**B**) healthy and fungal-infected samples. PC: principal component.

Principal component 1 (PC1) and PC2 explained 24.31% and 15.34% of the variation, respectively, as shown in Figure 7. Both Figure 7A,B are biplot scores of the same PCA, with (A) showing the genus (*Vand*a and *Cattleya*) differentiation and (B) the healthy and fungal-infected samples separation. In fact, only the healthy and fungal-infected separation was successful, mainly in the PC1. In this way, PLS-DA and OPS were applied to classify samples as healthy or fungal-infected to find the variables that contribute more to this separation.

**Figure 8:**
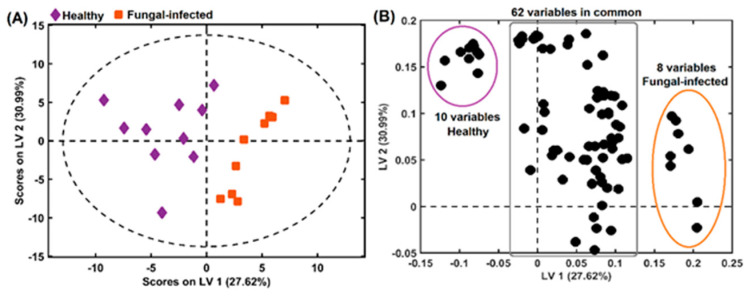
(**A**) Samples visualization in 2-D space using scores from PLS-DA obtained with OPS variable selection and (**B**) loadings from 80 variables selected to improve healthy and fungal-infected samples classification. LV: Latent variable.

Figure 8A shows the PLS-DA scores with two latent variables (LV), explaining 58.61% of the variance with the selected variables by OPS. In this case, with the selection of only 80 variables for both classes (healthy and fungal-infected), the separation was accentuated in LV1, with 27.62% of explained variance. These 80 variables can be divided into three groups: 62 common variables between healthy and fungal-infected samples, and 10 healthy and 8 fungal-infected variables, as shown in Figure 8B. For each group (healthy or fungal-infected samples), the most important variables were the 10 and 8 different variables highlighted in the loadings plot (Figure 8B). These 18 variables were previously described in Table 1.

In the healthy samples group, the most important selected variables were loliolide (*m*/*z* 196.110), 4-methoxy-3-methylbenzaldehyde (*m*/*z* 150.068), erianin (*m*/*z* 318.146), isorhamnetin 3-galactoside (*m*/*z* 478.111), isovitexin 2″-*O*-arabinoside (*m*/*z* 564.148), cinnamic alcohol (*m*/*z* 134.073), tricin 5-glucoside (*m*/*z* 492.127), isoshaftoside (*m*/*z* 564.148), acacetin (*m*/*z* 284.068), and allobetulinol (*m*/*z* 442.381). Some of these variables were detected in both groups; however, the variable selection appears only in the healthy samples group.

For the fungal-infected group, the following variables were founded: 3-[2-(3-hydroxyphenyl) ethyl]-5-methoxyphenol (*m*/*z* 244.109), galaxolidone (*m*/*z* 272.177), 3-(4-hydroxy-3-methoxyphenyl) propyl 3-(4-hydroxyphenyl) (*m*/*z* 330.147), salidroside (*m*/*z* 300.147), rutin (*m*/*z* 610.153), gigantol (*m*/*z* 274.121), isovitexin (*m*/*z* 432.105), and homoorientin (*m*/*z* 448.100). Most of these variables were described earlier in this study as detected only in fungal-infected samples.

In summary, our findings in chemometric data analysis were supported by the comprehensive structural annotation of the metabolites, being an essential tool for discovering antifungal compounds from the Orchidaceae family.

## 3. Materials and Methods

### 3.1. Chemicals and Materials

Acetonitrile, n-hexane, and methanol HPLC-grade were purchased from Tedia Company (Fairfield,OH, USA). Formic acid, caffeine-^13^C_3,_ and Supelclean C18 SPE cartridges (3 mL) were purchased from Sigma Aldrich (St. Louis, MO, USA). Progesterone-d_9_ was purchased from CDN Isotopes (Quebec, Canada). Ultrapure water was produced using a water purification system (Master System MS2000, Gehaka, São Paulo, Brazil) with a resistivity of 18.2 MΩcm.

### 3.2. Plant Material

Healthy and fungal-infected fresh leaves from five Vanda sp. and Cattleya sp. plants were collected from a private greenhouse in Lençois Paulista, São Paulo, Brazil (22°36′46.2″ S 48°50′02.1″ W). The plants were maintained at −80 °C until freeze-drying and sample preparation.

### 3.3. Sample Preparation

The fresh leaves were frozen at −80 °C and then freeze-drying for 72 h. The freeze-drying material was extracted with methanol. The solvent was removed using a Speedvac concentrator (Thermo Scientific Savant SPD131DDA). Subsequently, the extracts were submitted to a cleanup step (using SPE C18 cartridges) to eliminate chlorophyll and other interferences. The final extracted material (2 mL of methanol) was concentrated in a Speedvac concentrator. One milligram of dried extracts was made-up to 1 mL of methanol. This solution was diluted to 1:5 (v/v) in methanol, filtered (0.45 µm), and used in LC-MS analysis. Before the injections, the samples were spiked with a mixed standard solution (caffeine-^13^C_3_ and progesterone-d_9_, at 2.5 µg mL^−1^).

### 3.4. LC-HRMS Analysis

LC-HRMS/MS analyses were performed on an HPLC-UV 1220 Infinity II (Agilent Technologies) coupled with a Q-Exactive hybrid Quadrupole-Orbitrap high-resolution mass spectrometer (Thermo Scientific) as well as an electrospray ionization source. The column used in this study was an Infinity Lab Poroshell 120 EC-C18 column (4.6 × 100 mm × 2.7 μm Agilent). All samples were analyzed using a gradient elution program. The binary mobile phase comprised A (water with 0.1% formic acid) and B (methanol). The gradient elution started at 5% (B) and linearly increased to 100% (B) in 40 min and kept constant for 10 min at 100% (B). The eluent was restored to the initial conditions in 10 min. The flow rate was set at 0.3 mL min^−1^. The injection volume was 30 μL, and the column temperature was set at 35 °C. The electrospray ionization was operating with the following parameters: spray voltage 3.5 kV; capillary temperature: 269 °C; S-lens RF level 50 V; sheath gas flow rate at 53 L min^−1^; aux gas flow rate at 14 L min^−1^; sweep gas flow rate 3 L min^−1^. The high-resolution mass spectrometry (HRMS) was obtained under full MS/dd-MS^2^ mode. The mass range in the full MS scanning experiments was m/z 80–1200. The top 5 (TopN, 5, loop count 5) most abundant precursors were sequentially transferred for collision for fragmentation acquisition. The collision energy for target analytes was 20, 30, and 35 eV. Resolving power was set at 140,000 and 70,000 for full MS and dd-MS^2^ acquisitions, respectively.

### 3.5. Compound Characterization

The files acquired in the Q-Exactive hybrid Quadrupole-Orbitrap mass spectrometer for the methanolic extracts were converted from raw into (.mzML) format using MSConvert software (ProteoWizard, Palo Alto, CA, US) before being processed using MZmine software, version 2.53. We used metadata to organize compound information according to the Global Natural Products Social Molecular Networking (GNPS) online workflow (https://ccms-ucsd.github.io/GNPSDocumentation/ (accessed on 16 August 2022).

Metabolite annotations were based on searching the experimental spectra against the GNPS spectral library using the tools Classical Molecular Networking—MN [20], Feature-Based Molecular Networking—FBMN [33], DEREPLICATOR+ [21], Network Annotation Propagation—NAP [22], MOLDISCOVERY [23], MS2LDA [31], MolNetEnhancer [32], and analysis of chromatographic data such as retention time and UV spectra.

### 3.6. Chemometric Data Analysis

Twenty samples were used in data analysis: ten Vanda and ten Cattleya samples, using five healthy and five fungal-infected of each genus. The obtained .raw files were converted into .cdf format and imported to Matlab R2020a (Math Works, Natick, MA, USA), where all chemometric tools were applied.

An exploratory data analysis was carried out using principal component analysis (PCA) [50] to visualize the Vanda and Cattleya genus and healthy and fungal-infected samples in multidimensional space. PCA was carried out on the normalized and autoscale dataset with twenty samples and 1,021,894 variables (m/z 80 to 1200).

Additionally, partial least squares for discriminant analysis (PLS-DA) [51], a classification method based on PLS regression, was applied in healthy and fungal-infected samples. The ordered predictors selection (OPS) [52], a variable selection method adapted for classification, was applied to find the more essential and interpretative variables for healthy and fungal-infected classification.

## 4. Conclusions

In this study, we presented the application of the integrated metabolomics approach and the state-of-the-art computational tools to provide acute insights regarding the characterization of phytochemicals of healthy and fungal-infected leaves from *Vanda* and *Cattleya* species. The data analysis pipelines of untargeted metabolomics analysis enabled the characterization of a considerable variation in compounds belonging primarily to the class of flavonoids, phenolic acids, chromones, stilbenoids, tannins, terpenoids, and steroids. Flavonoids were identified as the major compounds in both species independent of the physiological condition (healthy or fungal-infected). The flavonoids afrormosin, acacetin, and stilbenoids were found chiefly in fungal-infected plants, while nitrogen compounds were found predominantly in samples from healthy plants. The tricin derivative flavonoid, and the loliolide terpenoid were found only in healthy plants. From the dereplication and in silico fragmentation tools, 202 and 145 unique metabolites were detected in healthy, and fungal-infected *Cattleya* leaves. For the genus *Vanda,* 166 and 146 unique metabolites were detected in healthy and fungal-infected leaves. The integrated metabolomics approach and the combination of structural annotation, data mining, and chemometric tools could be applied as a reference dataset for MS/MS-based untargeted metabolomic analysis of species from the Orchidaceae. In addition, based on the interesting results shown in the present study, the model data on the molecular annotation adopted here may be further explored with high efficiency through other species from the Orchidaceae family.

## Figures and Tables

**Table 1:** Results of the metabolite annotation from Orchidaceae species (*Vanda* and *Cattleya*) through LC-HRMS/MS analysis in positive ion mode ESI.

Genus	RT (Min)	Exact Mass	Molecular Formula	Metabolite Name	Chemical Class
*Vanda*	1.72	122.037	C_7_H_6_O_2_	4-Hydroxybenzaldehyde	Phenolic acid
*Vanda*	1.77	124.052	C_7_H_8_O_2_	3-hydroxybenzyl alcohol	Benzenoid
*Vanda*	24.22	134.073	C_9_H_10_O	Cinnamic alcohol	Phenylpropanoid
*Vanda/Cattleya*	2.02	150.068	C_9_H_10_O_2_	4-methoxy-3-methylbenzaldehyde	Benzenoid
*Vanda*	28.78	162.068	C_10_H_10_O_2_	4-Methylcinnamic acid	Phenylpropanoid
*Vanda*	1.72	164.047	C_9_H_8_O_3_	*p*-coumaric acid	Phenylpropanoid
*Cattleya*	12.53	168.042	C_8_H_8_O_4_	Benzoic acid, 2,4-dihydroxy-, methyl ester	Phenolic acid
*Vanda*	20.78	196.110	C_11_H_16_O_3_	Loliolide	Terpenoid
*Vanda*	29.26	210.089	C_11_H_14_O_4_	Sinapyl alcohol	Phenylpropanoid
*Vanda/Cattleya*	16.80	224.068	C_11_H_12_O_5_	Sinapic acid	Phenylpropanoid
*Vanda/Cattleya*	22.06	242.094	C_15_H_14_O_3_	Thunalbene	Stilbenoid
*Vanda/Cattleya*	29.32	244.109	C_15_H_16_O_3_	3-[2-(3-hydroxyphenyl)ethyl]-5-methoxyphenol	Stilbenoid
*Cattleya*	31.36	254.058	C_15_H_10_O_4_	Daidzein	Isoflavonoid
*Vanda*	27.28	256.074	C_15_H_12_O_4_	Liquiritigenin	Flavonoid
*Vanda/Cattleya*	33.93	258.125	C_16_H_18_O_3_	3′-*O*-Methylbatatasin III	Stilbenoid
*Vanda/Cattleya*		260.104	C_15_H_16_O_4_	Tristin	Stilbenoid
*Vanda/Cattleya*	30.11	260.105	C_15_H_16_O_4_	Dendrosinene B	Stilbenoid
*Vanda*	33.56	266.094	C_17_H_14_O_3_	4′-Methoxy-6-methylflavone	Flavonoid
*Vanda/Cattleya*	39.50	272.177	C_18_H_24_O_2_	Galaxolidone	Terpenoid
*Vanda/Cattleya*	28.65	274.121	C_16_H_18_O_4_	Gigantol	Stilbenoid
*Vanda/Cattleya*	32.23	274.121	C_16_H_18_O_4_	3-Methoxy-5-[2-(3-methoxyphenyl)ethyl]-1,2-benzenediol	Stilbenoid
*Cattleya*	31.93	284.068	C_16_H_12_O_5_	Acacetin	Flavonoid
*Vanda/Cattleya*	31.68	289.143	C_17_H_20_O_4_	3-*O*-Methylgigantol	Stilbenoid
*Cattleya*	34.35	298.084	C_17_H_14_O_5_	Afrormosin	Isoflavonoid
*Cattleya*	10.01	300.120	C_14_H_20_O_7_	Salidroside	Phenol
*Vanda*	36.41	300.136	C_18_H_20_O_4_	2,3,5,7-tetramethoxy-9,10-dihydrophenanthrene	Stilbenoid
*Vanda/Cattleya*	31.44	318.146	C_18_H_22_O_5_	Erianin	Stilbenoid
*Vanda*	31.22	330.147	C_19_H_22_O_5_	3-(4-hydroxy-3-methoxyphenyl)propyl 3-(4-hydroxyphenyl)propanoate	Phenol
*Cattleya*	26.91	348.209	C_24_H_28_O_2_	Bexarotene	Terpenoid
*Vanda/Cattleya*	22.81	374.230	C_19_H_34_O_7_	(2R,3S,4S,5R,6R)-2-(hydroxymethyl)-6-[4-(4-hydroxy-2,6,6-trimethylcyclohexen-1-yl)butan-2-yloxy]oxane-3,4,5-triol	Terpenoid
*Vanda/Cattleya*	17.70	389.217	C_19_H_32_O_8_	(2R)-4-[(1S)-1-Hydroxy-2,6,6-trimethyl-4-oxo-2-cyclohexen-1-yl]-2-butanyl beta-D-glucopyranoside	Terpenoid
*Vanda/Cattleya*	35.31	390.204	C_22_H_30_O_6_	7b,9-Dihydroxy-3-(hydroxymethyl)-1,1,6,8-tetramethyl-5-oxo-1,1a,1b,4,4a,5,7a,7b,8,9-decahydro-9aH-cyclopropa [3,4]benzo [1,2-e]azulen-9a-yl acetate	Terpenoid
*Vanda/Cattleya*	21.65	420.142	C_21_H_24_O_9_	Rhapontin	Stilbenoid
*Vanda*	16.08	432.105	C_21_H_20_O_10_	Isovitexin	Flavonoid
*Vanda/Cattleya*	12.84	432.163	C_19_H_28_O_11_	Darendoside A	Phenylethanoid
*Cattleya*	44.93	442.381	C_30_H_50_O_2_	Allobetulinol	Terpenoid
*Vanda*	16.61	448.100	C_21_H_20_O_11_	Homoorientin	Flavonoid
*Vanda*	22.65	464.095	C_21_H_20_O_12_	Hyperoside	Flavonoid
*Vanda*	24.66	478.111	C_22_H_22_O_12_	Isorhamnetin 3-galactoside	Flavonoid
*Cattleya*	29.47	492.127	C_23_H_24_O_12_	Tricin 5-glucoside	Flavonoid
*Vanda*	1.80	564.148	C_26_H_28_O_14_	Isoschaftoside	Flavonoid
*Cattleya*	21.31	564.148	C_26_H_28_O_14_	Isovitexin 2′’-*O*-arabinoside	Flavonoid
*Cattleya*	18.13	582.231	C_28_H_38_O_13_	2-[[5-(4-hydroxy-3,5-dimethoxyphenyl)-6,7-bis(hydroxymethyl)-1,3-dimethoxy-5,6,7,8-tetrahydronaphthalen-2-yl]oxy]-6-(hydroxymethyl)oxane-3,4,5-triol	Lignan
*Vanda/Cattleya*	15.80	594.158	C_27_H_30_O_15_	Saponarin	Flavonoid
*Vanda*	22.84	610.153	C_27_H_30_O_16_	Rutin	Flavonoid

## Data Availability

All support data used in this study are available from the authors.
